# Utilization of psychotropic medicines in Romania during 1998–2018

**DOI:** 10.3389/fphar.2023.1157231

**Published:** 2023-03-27

**Authors:** Irina Iaru, Camelia Bucsa, Andreea Farcas, Cristina Pop, Anamaria Cristina, Sebastian Armean, Irina Brumboiu, Oliviu Vostinaru, Cristina Mogosan

**Affiliations:** ^1^ Department of Pharmacology, Physiology and Pathophysiology, Faculty of Pharmacy, “Iuliu Hatieganu” University of Medicine and Pharmacy, Cluj-Napoca, Romania; ^2^ Pharmacovigilance Research Center, “Iuliu Hatieganu” University of Medicine and Pharmacy, Cluj-Napoca, Romania; ^3^ Department of Pharmacology, Toxicology and Clinical Pharmacology, Faculty of Medicine, “Iuliu Hatieganu” University of Medicine and Pharmacy, Cluj-Napoca, Romania; ^4^ Department of Epidemiology, Faculty of Medicine, “Iuliu Hatieganu” University of Medicine and Pharmacy, Cluj-Napoca, Romania

**Keywords:** drug utilization, psychotropic medicines, anxiolytics, antidepressants, antipsychotics

## Abstract

**Background:** Mental disorders can have a significant impact on patients’ life, including economic, social and individual consequences, and psychotropic medication is essential to treat these conditions. Psychotropic drug utilization studies contribute to a clearer picture of the management of these conditions. Data published from Romania on this topic is limited. The present study aims to characterize the utilization patterns of anxiolytics, antidepressants (ADs), and antipsychotics (APs) in Romania during 1998–2018.

**Methods:** Drug utilization data were provided by Management Center for Documentation, Information and Marketing (CEGEDIM) Romania and quantitative data for each psychotropic medicine were converted to total defined daily doses (DDDs) and to DDD/1000inhabitants/day (DDD/TID). The total use of medicines in DDD/TID was computed in order to obtain the drug utilization 90% (DU90%) segment.

**Results:** An increasing trend in total utilization of psychotropic medicines in Romania started in 2004. Anxiolytics use was predominant until 2013 and the yearly anxiolytic use over the entire study period remained between 10 and 15 DDD/TID. Diazepam lost popularity over time in detriment of the utilization of other anxiolytic benzodiazepines, such as alprazolam and lorazepam. ADs utilization markedly increased during the study period (the average annual growth rate was 13.66% starting 1999). Selective serotonin reuptake inhibitors (SSRIs) became present on the 2008 DU90% and was the dominant class of ADs, with sertraline being the most prescribed, followed by escitalopram and paroxetine. APs utilization showed an increasing trend from 2003 until 2018. Atypical APs became present on the 2008 DU90%, while typical APs were no longer included in the 2018 DU90%. Among atypical APs, olanzapine was the main agent prescribed, and starting 2010 was followed by quetiapine and risperidone. The uptake of APs long-acting formulations became more evident during the last analyzed years (2015–2018).

**Conclusion:** We observed an increasing utilization of APs and a more prominent increase in ADs utilization in Romania during 1998–2018. The anxiolytic prescribing remained nearly stable during this time. Further research can bring more information on the various factors influencing psychotropic utilization in Romania.

## 1 Introduction

Mental disorders have an enormous economic, social and individual impact in patients and therefore their prevention and treatment should represent a priority for healthcare systems. Nearly 84 million people were affected in 2016 across the European Union (EU), with more than one in six people (17.3%) having a mental problem: anxiety disorders were the most common (5.4%), followed by depressive disorders (4.5%); schizophrenic disorders affected 0.3% of the EU population. The estimated direct and indirect costs related to mental disorders were at more than 4% of gross domestic product (GDP) (The Organisation for Economic Co-operation and Development/European Union, 2018). In Romania, the national prevalence of mental disorders in 2016 was 14.3%; anxiety disorders were the most common (nearly 4%), followed by depressive disorders (nearly 3%). Schizophrenic disorders affected around 1% of the Romanian population (The Organisation for Economic Co-operation and Development/European Union, 2018). The estimated costs related to mental health problems were 2.1% of GDP (Iovu and Breaz, 2019).

Throughout time, the treatment of mental conditions has significantly evolved and psychotropic medicines have undergone various changes in use; in general, newer molecules have gain popularity, showing better efficacy and tolerability than traditional ones, new indications have expanded the use of some pharmacological classes [such as selective serotonin reuptake inhibitors (SSRIs), or antipsychotics (APs)], or safety issues have limited the use of some agents (such as agomelatine) (European Medicines Agency, 2014). Moreover, other factors such as available therapeutic guidelines and reimbursement levels have also significantly influenced the use of psychotropic medicines (Forns et al., 2019).

Previous studies have shown that psychotropic polypharmacy is a common practice in some Romanian hospitals (Jordanova et al., 2011), irregular antidepressant use was associated with the attitudes towards mental health disorders in Romanian patients (Lewer et al., 2015), or found benzodiazepines and antipsychotics among potentially inappropriate medications in older adults’ pharmacotherapy (Primejdie et al., 2016). However, published results on psychotropic drug utilization remain very scarce in Romania, but undoubtedly, they represent a necessity for a better understanding of the patterns of psychotropic drug utilization among patients. Especially when facing an upward trend in the prevalence of mental disorders (Ministry of Health et al., 2019). Therefore, the present study complements this limited drug utilization information, having the objective to determine and analyze the utilization patterns of anxiolytics, antidepressants (ADs), and antipsychotics (APs) in Romania during 1998–2018.

## 2 Materials and methods

### 2.1 General considerations for psychotropic prescribing in Romania

In Romania, psychotropic medication can be generally prescribed in outpatient care by specialists or family medicine (FM) physicians and in inpatient care by specialists. Psychiatrists are the main prescribers; however, other specialists (such as neurologists or cardiologists) can prescribe psychotropic medication. FM physicians have a gatekeeping role in primary care, although patients can directly access specialists. In order to assure reimbursement of psychotropic medication and patient accessibility within the social insurance health system, prescribers must follow national regulations and protocols published by the National Health Insurance House (NHIH) and have to use specific approved diagnostic codes for each psychotropic medication. Relevant psychotropic compounds are listed on the official national Reimbursement List, where they can be found with different reimbursement percentages, depending on criteria such as medicine status—innovative or generic, type of the targeted disease or available national health programs. Psychotropic medication requires a medical prescription in order to be dispensed from retail pharmacies. When a specialist prescribes the treatment during hospitalization, the medical prescription can be issued after discharge in certain situations by the FM physician if the patient needs to continue the treatment in outpatient care (European Observatory on Health Systems and Policies, 2016; The Government of Romania, 2018; European Observatory on Health Systems and Policies, 2021). Nevertheless, there are prescribing specifics which support the trend in psychotropic utilization in our study, aspects that are further discussed for each psychotropic class.

### 2.2 Data source

National Agency for Medicines and Medical Devices of Romania (ANMDMR) does not hold on its own a national database with drug consumption data, and, by national regulation, Management Center for Documentation, Information and Marketing (CEGEDIM) Romania is the provider of data consumption for the National Agency. CEGEDIM Romania is an innovative technology and services company in the field of digital data flow management for healthcare ecosystems. At the request of ANMDMR management, CEGEDIM provides information to enable activities such as monitoring the market of medicines in Romania, the share of pharmaceutical companies and the share of the main therapeutic groups on the Romanian medicine market, or providing synthetic information on the evolution of the pharmaceutical market in Romania. This is the main reason for including in our analysis CEGEDIM data, and another reason is the availability of data for a longer period of time, 21 years (1998–2018).

A retrospective descriptive study was conducted on estimated national sell-out data on psychotropic medicines for the 1998–2018 period. Data originated from the CEGEDIM Pharma and Hospital Report study and covered a panel of 4,700 retail and 75 hospital pharmacies, representing over 60% of retail and 16% of hospital pharmacies in Romania. The error margin (at 95% confidence level) for national data extrapolation was ±1% in retail and ±10.4% in hospital. The dispensing data provided by CEGEDIM were: Anatomical Therapeutic Chemical (ATC) code level 5, International Non-proprietary Name (INN), pharmaceutical form, strength (mg/dose), number of doses per package, and number of packages (units) dispensed each year. The analyzed therapeutic agents are listed in Table 1.

**TABLE 1:** Psychotropic medications considered for the analysis.

Anxiolytics		Antidepressants			Antipsychotics	
Benzodiazepines	Other	SSRIs^a^	Tricyclics/Tetracyclic	Other	Typical	Atypical
Alprazolam	Buspirone	Escitalopram	Amitriptyline	Agomelatine	Haloperidol	Tiapride
Lorazepam	Etifoxine	Paroxetine	Clomipramine	Trazodone	Levomepromazine	Sulpiride
Diazepam	Meprobamate	Sertraline	Doxepine	Bupropione	Zuclopenthixol	Amisulpride
Bromazepam	Hydroxyzine	Fluoxetine	Imipramine	Tianeptine	Flupentixol	Aripiprazole
Medazepam		Fluvoxamine	Nortriptyline		Trifluoperazine	Ziprasidone
Clobazam		Citalopram	Trimipramine		Thioridazine	Olanzapine
Oxazepam		SNRIs/NRIs^a^	Maprotiline		Chlorpromazine	Clozapine
Clordiazepoxide		Duloxetine	NaSSAs^a^		Thioproperazine	Paliperidone
Tofisopam		Venlafaxine	Mirtazapine		Fluphenazine	Quetiapine
Clorazepate dipotassium		Milnacipran	Mianserine		Droperidol	Risperidone
		Reboxetine			Pipotiazine	Loxapine
					Periciazine	Sertindole

^a^ SSRIs, selective serotonin reuptake inhibitors; SNRIs/NRIs, serotonin-norepinephrine reuptake inhibitors/norepinephrine reuptake inhibitors; NaSSAs, noradrenergic and specific serotonergic antidepressants.

### 2.3 Data analysis

Quantitative data for each medicine were converted to total defined daily doses (DDDs) and to number of DDD/1000inhabitants/day. DDD is the assumed average maintenance dose per day for a drug used for its main indication in adults and it was established by the World Health Organization (WHO) Collaborating Centre for Drug Statistics Methodology and extracted from WHO ATC/DDD Index (https:// www.whocc.no/atc_ddd_index/) (Salvesen Blix, 2016; World Health Organization, 2022). Total DDDs were computed for each available product of each INN, using the following formula: Total DDDs = (number of packages *number of doses per package *number of mg per dose)/WHO DDD (mg). We then obtained DDDs/1000inhabitants/day for each INN per year, using the formula: DDDs/1000inhabitants/day = (Total DDDs *1000)/(number of inhabitants in Romania for the year *days for the year). Number of inhabitants for the year was extracted from Eurostat database (European Commission, 2022). Hereafter, the short DDD/TID was used as reference to DDDs/1000inhabitants/day.

In order to assess drug prescribing over time for anxiolytics, ADs and APs, we applied drug utilization 90% (DU90%) method, which represents the number of medicines that account for the 90% of the total use. We considered the total use of medicines in DDD/TID and computed how many medicines accounted for the DU90% segment for 1998, 2008 and 2018 (Wettermark et al., 2003) (7).

## 3 Results

### 3.1 Total utilization of psychotropic medicines

**FIGURE 1:**
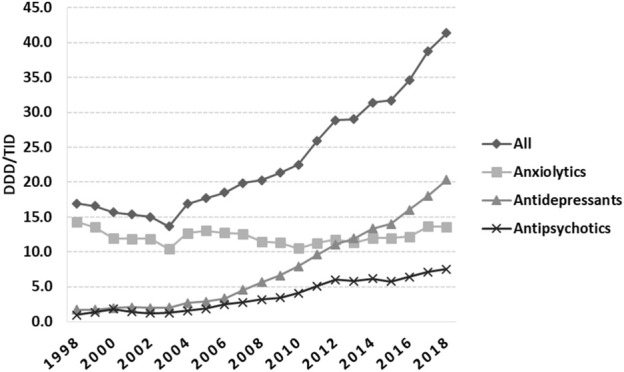
Total use of psychotropic medicines in Romania during 1998–2018, in DDD/TID. (DDD/TID, DDD/1000inhabitants/day).

An increasing trend in total utilization of psychotropic medicines in Romania started in 2004 (Figure 1), with anxiolytics being predominant until 2013, when ADs took the lead until 2018. During 1998–2003, the total utilization decreased by 19.47%, from 16.94 in 1998 to 13.64 DDD/TID in 2003, and then continuously increased until 2018 (41.37 DDD/TID).

### 3.2 DU90%

The DU90% profile for 1998 included 11 medicines, of which eight were anxiolytics and the other three were tricyclic antidepressants (ADs) (Table 2). Half of the psychotropic market was covered by diazepam. Diazepam remained on first position in 2008, covering 24.09% of the market, however, other anxiolytic benzodiazepines (BZDs) (alprazolam and lorazepam) were added to the list; also, three selective serotonin reuptake inhibitors (SSRIs) (sertraline, paroxetine, and escitalopram), other ADs (tianeptine, venlafaxine, mirtazapine, and mianserine), and four APs (olanzapine, risperidone, amisulpride, quetiapine, and haloperidol) were present in 2008 DU90%; tricyclic ADs were no longer present. The 2018 DU90% profile had alprazolam on the first place (13.07%), closely followed by sertraline (12.13%). In addition, of the total 16 molecules covered in 2018, the majority were ADs (*n* = 9), followed by anxiolytics (*n* = 4) and atypical APs (*n* = 3).

**TABLE 2:** DU90% profile of psychotropic medications in 1998, 2008 and 2018, expressed in DDD/TID.

1998				2008				2018			
RANK	Molecule	DDDs/TID	Share (%)	Rank	Molecule	DDDs/TID	Share (%)	Rank	Molecule	DDDs/TID	Share (%)
1	Diazepam	8.59	50.56	1	Diazepam	4.90	24.09	1	Alprazolam	5.50	13.07
2	Medazepam	2.05	12.08	2	Alprazolam	3.20	15.70	2	Sertraline	5.10	12.13
3	Meprobamate	1.42	8.38	3	Sertraline	1.46	7.17	3	Escitalopram	4.39	10.45
4	Hydroxyzine	0.68	4.02	4	Bromazepam	1.09	5.35	4	Lorazepam	3.46	8.24
5	Amitriptyline	0.62	3.63	5	Olanzapine	0.81	3.96	5	Diazepam	3.07	7.31
6	Chlordiazepoxide	0.49	2.90	6	Medazepam	0.76	3.75	6	Olanzapine	2.12	5.05
7	Imipramine	0.46	2.72	7	Paroxetine	0.73	3.57	7	Venlafaxine	1.86	4.43
8	Bromazepam	0.31	1.82	8	Lorazepam	0.70	3.42	8	Mirtazapine	1.86	4.43
8	Potassium clorazepate	0.23	1.34	8	Escitalopram	0.69	3.37	8	Paroxetine	1.70	4.05
10	Oxazepam	0.21	1.23	10	Tianeptine	0.59	2.89	10	Tianeptine	1.62	3.86
11	Doxepine	0.20	1.20	11	Venlafaxine	0.51	2.53	11	Quetiapine	1.58	3.76
				12	Meprobamate	0.49	2.41	12	Duloxetine	1.30	3.09
				13	Risperidone	0.43	2.09	13	Trazodone	1.27	3.03
				14	Mirtazapine	0.40	1.98	14	Bromazepam	1.08	2.57
				15	Amisulpride	0.39	1.93	15	Risperidone	1.06	2.53
				16	Quetiapine	0.36	1.79	16	Agomelatine	0.66	1.57
				17	Haloperidol	0.36	1.77				
				18	Mianserine	0.23	1.11				
				19	Potassium clorazepate	0.22	1.06				
	DU90%	15.26	89.88			18.31	89.98			37.65	89.58
	Others	1.72	10.12			2.04	10.02			4.38	10.42
	Total	16.98	100			20.35	100			42.03	100

DDD/TID, DDD/1000inhabitants/day.

### 3.3 Anxiolytics utilization

**FIGURE 2:**
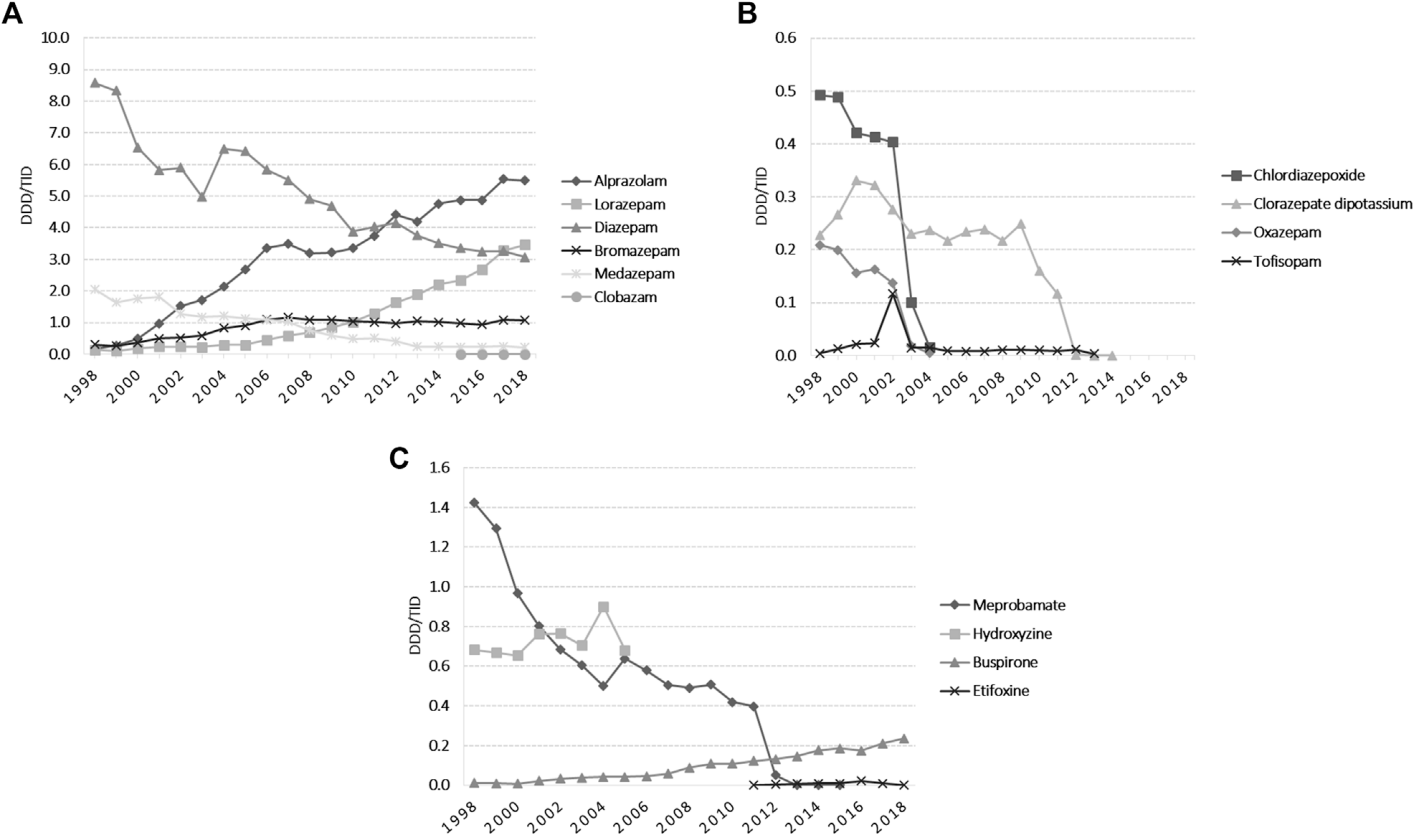
Anxiolytics utilization during 1998–2018 in DDD/TID. **(A)** BZDs for which the use was reported during 1998–2018. **(B)** BZDs for which the use stopped being reported during 1998–2018. **(C)** Use of other anxiolytics than BZDs. (DDD/TID, DDD/1000inhabitants/day; BZDs, benzodiazepines).

The total yearly anxiolytic use over the study period remained between 10 and 15 DDD/TID, with small fluctuations over time (Figure 1). Alprazolam, lorazepam and bromazepam use increased from 1998 until 2018. Alprazolam was the most prescribed anxiolytic starting with 2012, increasing 34.4-fold during the study period, and in 2018 was followed by lorazepam, which steadily increased between 1999 and 2018 (24-fold increase) (Figure 2A). Diazepam decreased 2.8-fold during 1998–2018, with fluctuations on this trend in 2004 and 2012. Medazepam was the second most used anxiolytic until 2002 and decreased thereafter to the fifth position in 2018. A very low yearly consumption of clobazam (<0.01 DDD/TID) was reported for the period 2015–2018. For other benzodiazepines (BZDs), their use stopped being reported during the study period, for oxazepam and chlordiazepoxide in 2004, for tofisopam in 2013 and for clorazepate dipotassium in 2014 (Figure 2B). Regarding non-BZDs anxiolytics, buspirone was the only one showing an increasing trend throughout the study period. Hydroxyzine and meprobamate had limited time of use, and etifoxine use started being reported in 2011 (Figure 2C).

### 3.4 Antidepressants (ADs) utilization

**FIGURE 3:**
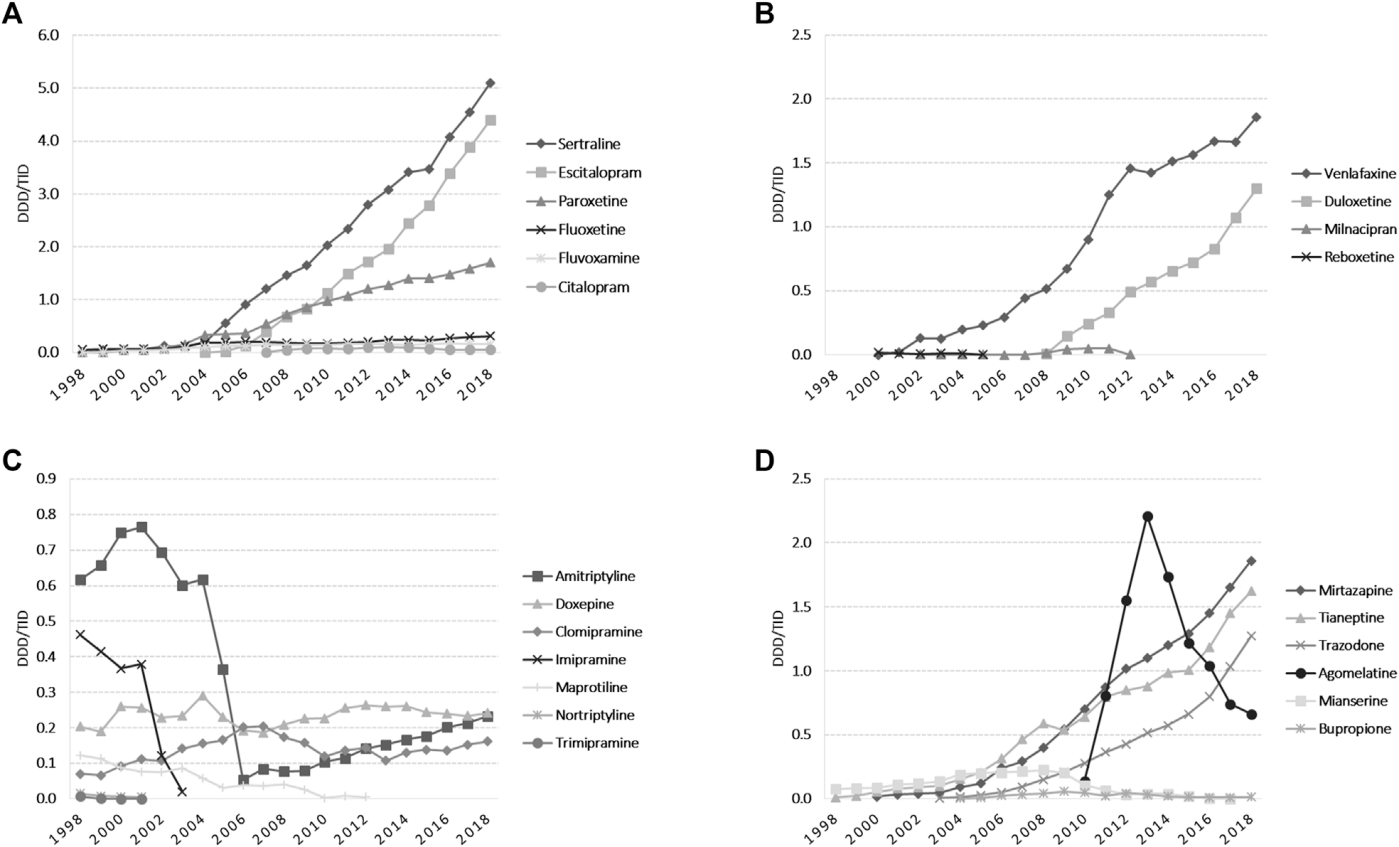
Antidepressants utilization during 1998–2018 in DDD/TID. **(A)** Use of SSRIs. **(B)** Use of SNRIs/NRIs. **(C)** Use of tricyclics/tetracyclic antidepressants. **(D)** Use of NaSSAs and other antidepressants. (DDD/TID, DDD/1000inhabitants/day; SSRIs, selective serotonin reuptake inhibitors; SNRIs/NRIs, serotonin-norepinephrine reuptake inhibitors/norepinephrine reuptake inhibitors; NaSSAs, noradrenergic and specific serotonergic antidepressants).

The total use of ADs markedly increased during the study period (20-fold, from 1.70 DDD/TID in 1998 to 20.30 DDD/TID in 2018) (Figure 1). The average annual growth rate starting 1999 was 13.66%. SSRIs was the dominant class of ADs (53.5% market share); lower increases were observed for other ADs classes (Figure 3). Among SSRIs, sertraline was the most prescribed (a 10-fold increase between 2005 and 2018), followed by escitalopram and paroxetine. A lower drug utilization during all years was noted for fluoxetine, fluvoxamine, and citalopram (Figure 3A). Tricyclic/tetracyclic ADs utilization remained low. Even if amitriptyline had the highest use among this ADs group during 1998–2004, it showed a 10-fold abrupt decrease from 2004 to 2006. Further on, amitriptyline use raised 4-fold from 2006 to 2018 (Figure 3C). Regarding SNRIs/NRIs group, venlafaxine use increased at the fastest pace, followed by duloxetine (Figure 3B). Among the NaSSAs and other ADs group, mirtazapine and trazodone use increased continuously until 2018. It was similar for tianeptine, excepting a slight decrease in 2009. Mianserine use was reported until 2017, with the highest use in 2008. In regards to other ADs, aglomelatine use increased 15-fold from 2010 to 2013, and then decreased sharply until 2018 (Figure 3D).

### 3.5 Antipsychotics (APs) utilization

**FIGURE 4:**
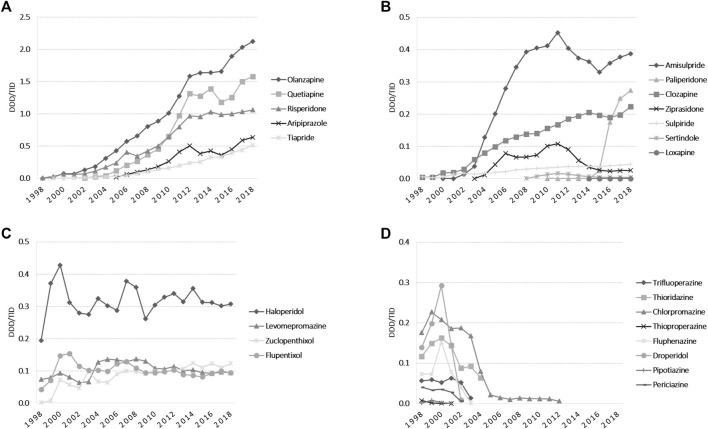
Antipsychotics use during 1998–2018 in DDD/TID. **(A)** Use of atypical APs, which in 2018 exceeded 0.50 DDD/TID. **(B)** Use of atypical APs, which in 2018 was under 0.50 DDD/TID. **(C)** Use of typical APs. **(D)** Typical APs for which use reporting discontinued during 1998–2018. (DDD/TID, DDD/1000inhabitants/day).

The total APs use slightly fluctuated in the early years, and then showed an increasing trend from 2003 (1.27 DDD/TID) until 2018 (7.50 DDD/TID). Among atypical APs, olanzapine remained the main agent used during all the evaluated period, and starting 2010 was followed by quetiapine and risperidone (Figure 4A). In 2018, amisulpride was followed by paliperidone and clozapine, with a reported use of less than 0.5 DDDs/TID. Paliperidone use increased more than 10-fold from 2015 to 2018 (Figure 4B). Regarding typical APs, we noticed small fluctuations in haloperidol use during 1998–2018, with the highest use in 2000. Levomepromazine, zuclopenthixole, and flupentixole use had slight fluctuations until 2008, and then remained mainly stable until 2018 (yearly use for each medication was under 0.15 DDDs/TID) (Figure 4C). Figure 4D presents the typical APs for which use reporting discontinued during the study period. When we analyzed the pharmaceutical forms, oral formulations of APs were predominant. For the injectable ones, we noticed a higher use of long-acting injectable APs than short-acting ones starting 2002. Long-acting formulations were constantly used during 2012–2015 and then increased 2-fold from 2015 (0.18 DDD/TID) to 2018 (0.38 DDD/TID). Short-acting APs use was stable during 2003–2009, showing afterwards an increasing use from 2010 (0.05 DDD/TID) to 2018 (0.18 DDD/TID).

## 4 Discussion

Romania can be found among the many countries where the stigma associated with mental illness and the belief that is better to simply avoid talking about these diseases could lead to lower prevalence among EU countries. Also, together with the great variability in the access to mental health services, the patients could be less likely to self-report mental illness and the diagnosis of mental disorders could be more difficult (The Organisation for Economic Co-operation and Development/European Union, 2018). Even so, in the present study we observed an increasing trend in the utilization of psychotropic medications in Romania during 1998–2018 and we discussed in the following sections for each therapeutic class potential factors that could have influenced the dynamics of the psychotropic pharmaceutical market.

### 4.1 Anxiolytics

The prescription and dispensing of BZDs is strictly controlled and regulated in Romania, they are listed on the national List of narcotic and psychotropic substances of medical interest, being legally classified as psychotropic. In the health insurance system, the pharmacy dispensing (based on a special green form retained upon release) has to be carried out within 30 days from the date of prescription; the physician can issue a new prescription before 30 days, for the same patient, if during the treatment there are changes in the patient’s state of health that require a change in the dose or medication, as well as when the prescribed quantity runs out. Anxiolytic therapy can be directly initiated by a FM physician for a period no longer than 30 days. After this period, specialist consult is necessary in order to continue the treatment. The FM physician can continue a treatment initiated directly by a specialist, based on the specialist’s medical letter (The Government of Romania, 2006; National Health Insurance House, 2018).

In both outpatient and inpatient care in Romania, anxiolytics are an important part of disease management protocols (e.g., locally established disease protocols in public hospitals) and of the common practice of physicians, regardless of specialization (e.g., in case of cardiovascular disease where anxiety and its associated disorders are common and may significantly influence cardiac health) (Balon et al., 2018).

The total yearly anxiolytics utilization in Romania between 10 and 15 DDD/TID during 1998–2018 was lower than in other European countries, such as Portugal, Spain or Luxembourg, which yearly exceeded 20 DDD/TID. The utilization however was similar in Sweden, Czech Republic or Norway (Estrela et al., 2020). We noticed a higher consumption than in Germany, where the guidelines on the treatment of anxiety disorders critically limit the use of BZDs because of their marked side effects (Ströhle et al., 2018).

The higher use of anxiolytics, mainly BZDs, during the first years of the study period (1998–2004), compared with the use of ADs, was a phenomenon similarly observed in Lithuania, and can be explained by the stigma associated with mental health disorders, amplified by the requirement for FP physicians to transfer patients to a psychiatrist if there was no clinical improvement. However, over time, once the awareness of mental disorders started to increase and SSRIs became more familiar among health authorities, professionals and patients, in parallel with increasing living standards in Romania, we observed a stabilization in total anxiolytic use, while the use of ADs markedly grew (Garuoliene et al., 2016).

A slight decrease in the use of anxiolytics has been observed across European countries, during a similar period (2000–2018), and could be explained by the trends in BZDs use and the awareness of their risks (such as dependence and withdrawal symptoms). This is especially the case of older adult patients (with co-morbidities and polypharmacy) who are the largest users of BZDs, often prescribed by non-psychiatrists (Guina and Merrill, 2018; Delaš Aždajić et al., 2019; Estrela et al., 2020).

Anxiolytic BZDs such as alprazolam and lorazepam seem however to have an increasing utilization trend in Romania, similar to other European countries (Torres-Bondia et al., 2020; Tavares et al., 2022). An explanation for this trend could be the fact that the therapy with these BZDs can be directly initiated by the FM physician. Also, their pharmacokinetic properties, with faster onset of action among BZDs, could make them preferable for prescribers or patients, especially when prescribed for insomnia treatment. In parallel, we noticed a decrease in the utilization of long half-life BZDs (such as diazepam), which could be explained by their limited use over time due to their risks (Guina and Merrill, 2018; Torres-Bondia et al., 2020).

There are no official national guidelines in Romania on how practitioners should prescribe BZDs. The prescription is usually guided by the available European guidelines or the clinical inertia in some clinical contexts. Nevertheless, many other factors can generally influence prescribing, but unfortunately, the available data did not permit such analysis (Riemann et al., 2017; Guina and Merrill, 2018; Ströhle et al., 2018; Delaš Aždajić et al., 2019).

For the other anxiolytics, buspirone slowly became more prescribed during the last years of our study. It is known for inducing less sedation than BZDs, with lower risk of tolerance, dependence or withdrawal problems (Wilson and Tripp, 2022). However, the profile of indications could be different from BZDs because buspirone takes 1–2 weeks of treatment for the installation of its therapeutic effects.

### 4.2 Antidepressants (ADs)

In general, in Romania, the ADs therapy can be initiated by a psychiatrist and continued by the psychiatrist or by the FM physician for a 3–6 months period based on a medical letter provided by the psychiatrist. Variations can appear here also, an example is the case of trazodone, for which the treatment can be initiated by the FM physician in case of a mild depressive episode, mild anxiety disorders or non-organic insomnia, with the evaluation of the risk-benefit ratio. After the first month of treatment, if the patient’s condition has not improved, the FM physician has the obligation to request a specialist consultation for clinical and therapeutic reevaluation. If the patient’s condition has improved, the FM physician can continue the prescription in the health insurance system for a maximum of 2 months (3 months in total). In case of tianeptine, the treatment can be initiated by the FM physician for the indication of anxious depressive disorder. Other particular cases are duloxetine, which can be prescribed also by specialists in neurology and/or diabetes, nutrition and metabolic diseases and/or with competence/certificate in diabetes (for pain management in case of diabetic neuropathy) or clomipramine—approved for the treatment of chronic pain conditions (National Agency of Medicines and Medical Devices in Romania, 2022; The Government of Romania (2006); National Health Insurance House (2018).

Despite the increasing evidence of efficacy and awareness for non-pharmacological interventions for depression and anxiety, ADs utilization seems to continuously escalate. The increasing prevalence, recognition and public awareness of depression and anxiety disorders, and generally of mental disorders, and the improved diagnosis of these conditions might contribute to this increase. Additionally, the number of therapeutic indications changed over time, at present ADs being also used as first-line treatment in anxiety disorders, panic attacks, agoraphobia and post-traumatic stress disorder (Stephenson et al., 2013; Estrela et al., 2020). The prominent increase of ADs utilization in the present study was largely driven by rises in the utilization of SSRIs, but also of other ADs, such as SNRIs and NaSSAs. The observed trends are similar with other European studies showing increasing prescribing of ADs during the last years (Gusmão et al., 2013; Abbing-Karahagopian et al., 2014). But while other European countries reported in 2018 a utilization of ADs higher than 30 DDD/TID and reaching 110 DDD/TID in Portugal, ADs utilization in Romania remained much lower (20.30 DDD/TID in 2018) (Estrela et al., 2020). A study conducted in different European countries (using different data type and source) showed that in Romania ADs utilization in 2004 was 2.59 DDD/TID, which increased to 6.09 DDD/TID in 2009 (Gusmão et al., 2013).

The Romanian national guideline available on the treatment of depressive disorders dates from 2010, with an update in 2014 (Udriștoiu et al., 2010); Romanian Health Minister (2018). The financial support from national health insurance and the possibility of the FM physicians to initiate the therapy for some ADs highly influence the prescription choice. Even if the efficacy of ADs cannot be clearly differentiated among the available classes, the better tolerability of SSRIs, the once-daily administration, and the less dose-titration needed as compared to TCAs explain their increasing uptake over time (Stephenson et al., 2013).

Sertraline and escitalopram had the highest utilization among SSRIs; their higher efficacy and safety as compared to most other SSRIs support this observation (Forns et al., 2019). We observed no changes in the increasing trend of escitalopram utilization in Romania, after its authorization in 2003, once generic citalopram (authorized in 2006) or other generic SSRIs became available at lower costs for the healthcare system, as it was seen in Scotland. The demand-side measures implemented in Scotland (such as limiting the prescribing of escitalopram to less than 5%–10% of all SSRI prescriptions) led to a lower utilization of escitalopram, and to decreased pharmaceutical expenditure for the health system, even the SSRIs utilization increased in volume during 2001–2007. The pattern observed in the utilization of escitalopram in Romania suggests that there were no demand-side measures implemented in Romania to counteract pharmaceutical company marketing activities for limiting the prescribing of patented escitalopram, situation similar to Ireland and Portugal where appreciably increased expenditure was also noticed (Godman et al., 2013a). Unfortunately, expenditure data was not available for the present analysis. The concerns with possible QTc interval prolongation with citalopram and escitalopram were pointed out in 2011 by the European Medicines Agency (EMA) and resulted in update of the Prescription Information and Summary of Product Characteristics for citalopram and escitalopram and issued recommendations regarding dose reduction, contraindications, precautions, interactions and alternative SSRIs (such as sertraline) for new patients. The warning contributed to their falling prescribing during 2007 and 2017 in Scotland. In our study we did not observed changes in the ascending prescribing trend of escitalopram and citalopram, which might have been caused by the safety issues with potential impact on patients’ health state (Godman et al., 2019).

Duloxetine use started being reported in our study in 2008 with an increasing prescribing trend since then. It is also authorized for the treatment of diabetic neuropathy. Its use came in second position after venlafaxine for the SNRIs/NRIs group. The situation was different in Sweden, where prescription restrictions for duloxetine were introduced in July 2010 due to its weak effectiveness as compared to other ADs, and limited its use in patients with depression or general anxiety disorders who have been prescribed at least two other ADs without reaching their treatment goals. This consequently determined significant grow in the utilization of venlafaxine in Sweden (Godman et al., 2013c).

We could see that in Romania amitriptyline lost its popularity at the time of the increasing uptake of new SSRIs, in particular with the start of escitalopram uptake in 2004. However, its utilization in monotherapy in the management of neuropathic pain and other conditions, such as migraine, stress, and sleep disorders, and the rich clinical experience support its use even after 2004 (Forns et al., 2019).

Regarding agomelatine, after its approval in 2009, a considerable level of non-compliance with the recommended liver monitoring program was observed across EU, which led in 2014 to a need to reiterate its importance (liver function monitoring before the initiation of treatment and regularly during the treatment, at 3, 6, 12, and 24 weeks and thereafter when needed). Agomelatine still remained with a positive benefit-risk balance, however, we observed in this study a high reduction in its use over time in Romania (European Medicines Agency, 2014).

### 4.3 Antipsychotics (APs)

Generally, in Romania, the APs therapy can be initiated by a psychiatrist, and can be continued, depending on the product formulation, either by the psychiatrist or by the FM physician. For example, in the case of olanzapine, after the therapy initiation, the prescription for oral treatment can be continued by the FM physician for a 3–6 months period based on a medical letter provided by the psychiatrist (same for oral risperidone, aripiprazole and paliperidone). For the parenteral formulations, the olanzapine therapy continues only by the decision of the psychiatrist, and the depot products can be administered only in a specialized health unit, with patient monitoring for 3 h post-injection. There can be other specialists prescribing APs, such example is quetiapine or clozapine, which can be prescribed by neurologists for psychotic disorder associated with Parkinson disorder (The Government of Romania, 2006; National Health Insurance House, 2018).

The increasing utilization trend of APs has been observed during the last decade across European countries. In Romania, the utilization seemed to be higher than in Albania, for example, but lower than Estonia, Finland or Norway (Halfdanarson et al., 2017; Forns et al., 2019; Kakariqi and Vyshka, 2020; Taipale et al., 2020). Official national guidelines for the treatment of schizophrenia date from 2010, with an update in 2014 Romanian Health Minister (2018). APs are primarily approved for the treatment of severe psychiatric disorders, such as schizophrenia and bipolar mania. However, they can be prescribed in the case of various other psychiatric disorders, including major depressive disorder, substance abuse, anxiety disorders, sleep disorders, autism spectrum disorders or aggression and behavioral symptoms of dementia in elderly. These expanded approved indications and even the increasing off-label use of APs could also explain their rise in utilization (Stephenson et al., 2013; Forns et al., 2019). On the other side, for some compounds, such as thioridazine, severe side effects supported the withdrawal of the marketing authorization in 2005 (severe cardiac arrhythmias) (McNaughton et al., 2014).

Other European drug utilization studies showed that the increasing use of APs was mainly attributed to increases in olanzapine and quetiapine use, both of which were the most commonly used APs after 2010 in our study too (Polić-Vižintin M, 2016; Hojlund et al., 2019). We noticed the decline in the utilization of typical APs in favor of atypical APs. Atypical APs usually represent the first-line treatment of schizophrenia, due to their efficacy in controlling positive and negative symptoms, as compared to the typical APs, which mainly control positive symptoms and have a higher risk of extrapyramidal effects and tardive dyskinesia with long-term use (Meltzer and Gadaleta, 2021). This tendency was supported by increasing reimbursement of the newer generation of atypical APs, while the typical ones were withdrawn from the reimbursement list (National Health Insurance House, 2018).

There were only small fluctuations regarding the ascending trend of antipsychotic utilization in Romania, namely, for olanzapine, risperidone, quetiapine, and aripiprazole. However, for the 2012–2015 period, a slowdown of the upward utilization slope was observed. In 2012, electronic prescribing was introduced in Romania for all reimbursed medicines with the aim of helping improve monitoring the use of medicines and contribute to better prescribing practices (European Observatory on Health Systems and Policies, 2016). During 2012–2013, we can notice a small increase in olanzapine prescribing, while for the other three agents (risperidone, quetiapine, and aripiprazole) there was a small decrease in utilization. There was an extended inclusion of generic medicines for olanzapine on the reimbursement national List in 2012, as compared to the 2011 List, which could be responsible for this change. The decrease in co-payment for olanzapine products can support its increasing prescribing. At this time, risperidone generics were already available and present on the C1-G15 reimbursement list. During 2013–2014 a small increase in utilization of all four agents can be observed, but afterwards, during 2014–2015, there was again a small decrease in the utilization of risperidone, quetiapine, and aripiprazole. What happened in 2014 in Romania, was the introduction of paliperidone on the reimbursement List.

The situation is Romania could be different from what happened in Poland where there is a reference price system based on the class of medicine (Wladysiuk et al., 2011). Therefore, when generic olanzapine became available in Poland in 2003, it determined reduced patient co-payments for olanzapine treatment and appreciably increasing utilization, olanzapine becoming the atypical antipsychotic of choice prescribed starting 2003. However, when the branded generic risperidone formulations became available, due the supply problems with the first branded generic risperidone and limited access, patients had to cover the additional co-payment for the originator risperidone if the physician wished to prescribe risperidone rather than olanzapine, which determined stabilization in the risperidone utilization.

Depot formulations could be advantageous for increasing adherence to treatment, one of the main challenges at present of the schizophrenia treatment (Stephenson et al., 2013; Correll et al., 2016; Sultana et al., 2019; Orrico-Sanchez et al., 2020). Prescribing restrictions for long-acting antipsychotic injections could contribute to their limited use and slow uptake in Romania. For example, for risperidone the long-acting injectable treatment is reimbursed in Romania only in case of maintenance treatment for schizophrenia, primarily, or secondarily for bipolar disorders. There is a limited number of administrations/month accepted and only the specialist in psychiatry can continue prescribing the treatment after therapy initiation, and not the FP physician. Prescribing restrictions also present in Belgium contributed to a decreasing utilization of these products during 2011–2012 and was expected to be the same afterwards (Godman et al., 2013b). In Romania, the utilization trend was however with an upward slope, with a mainly stable use during 2012–2015 and a 2 fold increase from 2015 to 2018.

### 4.4 Strengths and limitations

The ATC/DDD methodology was valuable to our study, however, the aggregated utilization and the results presented in DDD/TID provided only a rough estimate of psychotropic utilization in Romania. Even if the utilization of psychotropic medication could be influenced by various factors, the results are of great importance for the drug utilization research field in Romania. Not only was the evolution of utilization for the three classes underlined, but also the main compounds that have driven the fluctuations in the pharmaceutical market over time and some possible explanatory factors. Comparison in drug utilization with different European countries was possible, but the results should be cautionary interpreted (e.g., due to differences in national guidelines recommendations, in prescription practices, availability of different medicines and formulations).

Regarding the CEGEDIM method limitations, we have to mention that in general, products with uneven distribution (e.g., niche products, products dispatched only in some pharmacies) may have had higher error margins. Also, new launched products may have had atypical sales pattern due to supply chain phenomena. No information was available on the profile of hospitals covered by CEGEDIM data (e.g., the percentage of psychiatric hospitals) or the characteristics of retail pharmacies dispensing the psychotropic medicines to patients, which could have had also an influence on the study results. Prescriber’s characteristics, clinical inertia or popularity of one medicine over the other due to marketing strategies of pharmaceutical companies may play an important role in medication prescribing in Romania, but information on these practices is not available in the present study (Bucsa et al., 2021). Moreover, reimbursement data would be essential to better characterize the prescribers, the patients, the indications, and how different reimbursement factors influence the psychotropic utilization.

## 5 Conclusion

We observed an increasing trend in the utilization of psychotropic medications in Romania during 1998–2018. While the anxiolytic drug prescribing in Romania remained nearly stable during this time, there was an increasing utilization of APs and a more prominent increase in ADs use. Short-acting BZDs gained ground to the detriment of long-acting BZDs. SSRIs use highly increased in parallel with the decline of TCAs. Atypical APs showed increasing utilization and the increasing uptake of depot formulations could offer important advantages to schizophrenic patients. Nevertheless, the results of this study show similarities with the psychotropic utilization in other European countries. At the same time, Romania becomes present on the international map of drug utilization studies for psychotropic medication, with available data for further cross-national comparisons. More in-depth studies are necessary to identify, describe and understand various factors influencing psychotropic utilization in Romania.

## Data Availability

The data analyzed in this study is subject to the following licenses/restrictions: Data was provided by CEGEDIM Romania. Requests to access these datasets should be directed to cazacuirina16@gmail.com.
